# Geographical proximity and the transmission of drug abuse among siblings: evaluating a contagion model in a Swedish National Sample

**DOI:** 10.1017/S2045796019000453

**Published:** 2019-08-23

**Authors:** Kenneth S. Kendler, Henrik Ohlsson, Alexis C. Edwards, Jan Sundquist, Kristina Sundquist

**Affiliations:** 1 Virginia Institute for Psychiatric and Behavioral Genetics, Virginia Commonwealth University, Richmond, VA, USA; 2 Department of Psychiatry, Virginia Commonwealth University, Richmond, VA, USA; 3 Department of Human and Molecular Genetics, Virginia Commonwealth University, Richmond, VA, USA; 4 Center for Primary Health Care Research, Lund University, Malmö, Sweden; 5 Department of Family Medicine and Community Health, Department of Population Health Science and Policy, Icahn School of Medicine at Mount Sinai, New York, USA; 6 Department of Functional Pathology, Center for Community-based Healthcare Research and Education (CoHRE), School of Medicine, Shimane University, Shimane, Japan

**Keywords:** Drug abuse, epidemiology, social contagion, spatial clustering

## Abstract

**Aims:**

Can drug abuse (DA) be transmitted psychologically between adult siblings consistent with a social contagion model?

**Methods:**

We followed Swedish sibling pairs born in 1932–1990 until one of them, sibling1 (S1), had a first DA registration. We then examined, using Cox regression, the hazard rate for a first registration for DA in sibling2 (S2) within 3 years of a first DA registration in S1 as a function of their geographical proximity. We examined 153 294 informative pairs. To control for familial confounding, we repeated these analyses in sibships containing multiple pairs, comparing risk in different siblings with their proximity to S1. DA was recorded in medical, criminal or pharmacy registries.

**Results:**

The best-fit model predicted risk for DA in S2 as a function of the log of kilometres between S1 and S2 with parameter estimates (±95% confidence intervals) of 0.94 (0.92; 0.95). Prediction of DA included effects of cohabitation and an interaction of proximity and time since S1 registration with stronger effects of proximity early in the follow-up period. Proximity effects were stronger for smaller S1–S2 age differences and for same- *v*. opposite-sex pairs. Sibship analyses confirmed sibling-pair results.

**Conclusions:**

Consistent with a social contagion model, the probability of transmission of a first registration for DA in sibling pairs is related to their geographical proximity and similarity in age and sex. Such effects for DA are time-dependent and include cohabitation effects. These results illustrate the complexity of the familial aggregation of DA and support efforts to reduce their contagious spread within families in adulthood.

Drug use and drug abuse (DA) often clusters in social and spatial networks (Galea *et al*., 2004; Dishion and Dodge, 2006; Christakis and Fowler, 2008; Rosenquist *et al*., 2010). Two mechanisms are commonly proposed to explain spatial clustering: correlated exposures to psychological or social risk factors and person to person transmission (Cheng *et al*., 2014) sometimes called ‘social contagion’ (Christakis and Fowler, 2013). DA strongly aggregates within families, likely as a result of genetic and shared environmental factors (Bierut *et al*., 1998; Merikangas *et al*., 1998). We have recently examined DA among parent–offspring, sibling and cousin pairs in Sweden showing stronger transmission in those living together *v*. those residing in the same town which in turn was stronger than those living only in the same large metropolitan area (Kendler *et al*., 2019). However, we are unaware of prior efforts to examine, using precise measures of geographical proximity, whether the transmission of DA within adult relatives contributes to this aggregation.

We here report a study building on our earlier results by utilising information in Sweden on the geographical location of all individuals' residence known to within 250 m. While our earlier report on DA was restricted to residences within the same metropolitan area, the current analyses follow-up sibling pairs throughout the country of Sweden. Specifically, we follow full-sibling pairs until one of them (S1) is first registered for DA. We then examine the hazard rate in the other sibling (S2) for DA over the next 3 years as a function of the geographical proximity of S2 to S1. We also examine whether the effect of proximity on risk for DA in S2 risk attenuates over time.

The validity of our approach towards elucidating the psychological transmission of DA is supported by evidence in adult siblings that proximity is related both to frequency of contact (Lee *et al*., 1990; White and Riedmann, 1992; Stocker and Lanthier, 1997; White, 2001; Spitze and Trent, 2006) and emotional closeness (Suggs, 1989; Lee *et al*., 1990; White and Riedmann, 1992; White, 2001; Spitze and Trent, 2006; Van Volkom, 2006; Mulder and van der Meer, 2009). Our second design examines sibships with multiple siblings. In them, we explore, after a first registration for DA in one sibling (S1), whether the risk among the remaining siblings (S2, S3, S4 etc.) is predicted by their geographical proximity to S1.

## Methods

This study utilised several Swedish population-based registers with national coverage, the availability and content of which have been described previously (Kendler *et al*., 2013*a*). Records are linked using unique personal identification numbers, replaced by anonymous serial numbers to maintain confidentiality. The study was approved by the Regional Ethical Review Board of Lund University. From the Swedish Multigenerational register, we included in the study database all possible full-sibling pairs, where both individuals within the pair were born between 1932 and 1990, and had a maximum age difference of 10 years. For pairs born prior to 1960, we required that both individuals were alive at 1985, and for individuals born 1960 and onwards we required that both were alive at age 15. The restriction that both siblings should be alive at age 15 was set because this is the age when individuals can be registered for DA in the criminal registers. The restriction that individuals born prior to 1960 also should be alive at age 25 is due to the fact that most of the registrations for DA occur before age 30 and the ascertainment for DA in the relevant registers are probably less complete during the 1970s and 1980s.

For all individuals, we included yearly information, from 1975 to 2012, about the place of residence within 250 m. This enabled us to examine the distance between places of sibling residence. DA was assessed in the Swedish Inpatient Register from 1973 to 2012, the Swedish Outpatient Register from 2001 to 2012, the Swedish Mortality Register (1969–2012), the Swedish Pharmacy Register (2005–2012), the Swedish Criminal (1973–2012) and Suspicion Registers (1998–2012). Specifically, DA was identified in the Swedish Medical and Mortality Registries by ICD codes (ICD8: Drug dependence (304); ICD9: Drug psychoses (292) and Drug dependence (304); ICD10: Mental and behavioural disorders due to psychoactive substance use (F10–F19), except for those due to alcohol (F10) or tobacco (F17)); in the Suspicion Register by codes 3070, 5010, 5011 and 5012, that reflect crimes related to DA and in the Crime Register by references to laws covering narcotics (law 1968:64, paragraph 1, point 6) and drug-related driving offences (law 1951:649, paragraph 4, subsection 2 and paragraph 4A, subsection 2). DA was identified in individuals (excluding those suffering from cancer) in the Prescribed Drug Register who had retrieved (in average) more than four defined daily doses a day for 12 months from either of hypnotics and sedatives (Anatomical Therapeutic Chemical (ATC) Classification System N05C and N05BA) or opioids (ATC: N02A).

We selected sibling pairs where at least one in the pair, sibling1 (S1), was registered for DA for the first time at which point follow-up began for sibling2 (S2) until registration for the specific phenotype, death, emigration or end of follow-up (3 years after S1's registration). We utilised a 3-year follow-up period because prior analyses identified elevated rates of DA registration for that period after a DA registration in a close relative (Kendler *et al*., 2019).

We used Cox proportional hazards model where the main predictor was distance in kilometres between siblings at S1's registration. We tested several models that allowed the relationship between distance and the outcome variable to differ and included or excluded an extra effect if S1 and S2 cohabitated. In all models age at registration in S1, age difference between S1 and S2 and small areas for market statistics (SAMS) density were included. The models we tested included, for example, the crude distance, the natural log of distance and models with a spline at different distances. The spline models suggest that there is one effect during the first kilometres and after a predefined ‘knot’ the effect will change. We included models where we placed the ‘knot’ at 5, 10, 15, 20 and 25 km. The models controlled for age in S2 at S1's registration, S1–S2 age differences, gender composition and SAMS *density* which reflected, within the SAMS where S2 resided at S1's registration, the proportion of DA registrations in individuals with ±5 years age difference to S2 within a 3-year interval around that registration. We included SAMS density because of prior evidence from multi-level modelling analyses of substantial concentration of DA in certain SAMS. Specifically, in Sweden SAMS units accounted for 4.5% of the population variance for DA (Kendler *et al*., 2015*b*).

Defined by Statistics Sweden to represent geographically distinct communities, there are ~9200 SAMS in Sweden, each with an average population of 1000. For the analysis of sibships, we included, in the Cox proportional hazards model, a separate stratum for each sibship that allows each sibship to have a separate baseline hazard function. The best fitting models were chosen by Akaike's information criterion (AIC) (Akaike, 1987).

We investigated the proportionality assumption by including an interaction term between log of time and proximity. This model was also used to calculate the hazard ratios (HR) at different time point during our follow-up period. Statistical analyses were performed in SAS 9.4 (SAS Institute, 2012).

## Results

We identified 153 294 sibling pairs informative for DA proximity analyses (Table 1). Within 3 years of the first DA registration of S1, 3.4% of S2s also had first registrations for DA. The mean distance between concordant and discordant siblings was, respectively, 44.8 and 76.4 km. The DA density in the SAMS region in which S2 was residing was higher when S2 was *v*. was not registered for DA (3.2 *v*. 1.8%).

**Table 1.** Descriptive statistics on full sibling pairs from the Swedish population where at least one in the pair are registered for DA

	DA				
	All	Male to male	Female to female	Female to male	Male to female
*N* pairs	153 294	54 163	21 149	21 325	56 657
% in S2 (within 3 years)	3.4	6.1	1.8	3.4	1.4
S2 affected					
Distance (km) 25th-50th-75th	0-2-18	0-1-12	1-7-39	1-6-44	0-4-30
Distance (km) mean	44.8	35.5	64.7	66.0	54.8
Log of distance 25th-50th-75th	0-0.6-2.9	0-0-2.5	0-1.8-3.7	0-1.7-3.8	0-1.1-3.4
Log of distance mean	1.51	1.25	2.08	2.08	1.81
Same household	34.7%	40.3%	20.5%	22.1%	29.6%
Age at registration S1	24.3	23.8	25.5	24.9	25.0
Age difference	3.4	3.4	3.4	3.4	3.5
YoB S1, mean	1979	1979	1977	1979	1978
YoB S2, mean	1979	1980	1977	1978	1979
SAMS density S1 (±5)	3.4%	3.5%	3.3%	3.5%	3.2%
SAMS density S2(±5)	3.2%	3.0%	3.2%	3.1%	3.0%
S2 not affected					
Distance (km) 25th-50th-75th	2-10-62	1-7-44	3-15-86	2-14-84	2-9-61
Distance (km) mean	76.4	67.5	88.1	87.1	76.2
Log of distance 25th-50th-75th	0.1-2.2-4.1	0-1.8-3.8	0.8-2.7-4.4	0.7-2.6-4.4	0.1-2.2-4.1
Log of distance mean	2.35	2.12	2.68	2.64	2.32
Same household	15.7%	19.6%	9.5%	10.9%	16.2%
Age at registration S1	29.4	28.9	31.2	31.2	28.6
Age difference	4.1	4.1	4.1	4.1	4.1
YoB S1, mean	1972	1973	1970	1970	1974
YoB S2, mean	1971	1972	1969	1969	1973
SAMS density S1 (±5)	2.4%	2.5%	2.2%	2.1%	2.6%
SAMS density S2(±5)	1.8%	1.9%	1.5%	1.5%	1.9%
Same area after 3 years	58%	58%	61%	61%	56%

DA, drug abuse; S1, Sibling 1; S2, Sibling 2; SAMS, Small Area for Market Statistics; YOB, year of birth.

**Fig. 1.**
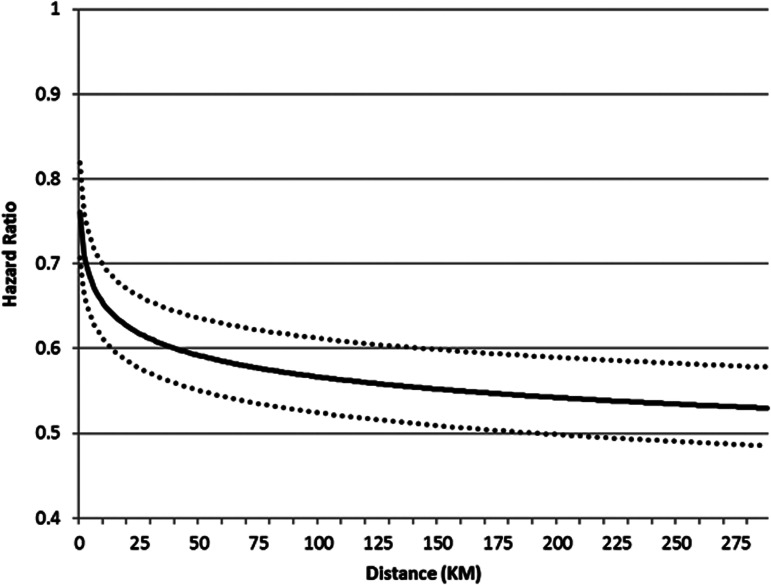
The HR (±95% CI) for a first registration of DA in S2 in 3 years after a first registration for DA in S1 as a function of distance (in kilometres) they were residing apart at the time of S1's DA. The HR does not begin at unity because of the cohabitation effect which is significant in this model. For details and covariates, see Table 3.

Controlling for SAMS density of DA, age difference and age at S1's DA registration, the best fit model for all sibling pairs included a household and distance effect (Table 2). Residing outside S1's household had a substantial protective HR for S2: 0.76 (0.71; 0.82). The estimated HR per log of kilometres for DA in S2 was 0.94 (0.92; 0.95) (Table 2, Fig. 1). The risk of DA in S2 fell rapidly as a function of distance for those living within 75 km of S1, then declined at a slower pace nearly asymptoting at ~150 km.

**Table 2.** Model fit for different model. Values are for AIC^a^

	DA				
Effects	All	Male to male	Female to female	Female to male	Male to female
SAMS density, age difference and age at registration	119 959.17	69 324.411	7295.922	13 913.470	16 629.771
Household	119 794.17	69 231.938	7297.090	13 915.304	***16 619.040***
Linear (distance)	119 884.12	69 269.884	7296.710	13 914.478	16 627.659
Linear (distance) + household	119 762.27	69 206.384	7298.208	13 916.444	16 619.497
Linear (distance) + quadratic (distance)	119 864.78	69 252.355	7298.179	13 916.428	16 629.674
Linear (distance) + quadratic (distance) + household	119 757.33	69 199.447	7299.822	13 918.406	16 621.149
Spline (Distance) with knot at 5 km	119 759.28	69 194.985	7298.095	13 915.836	16 623.381
Spline (distance) with knot at 5 km + household	119 741.78	69 190.501	7300.034	13 917.562	16 621.357
Spline (distance) with knot at 10 km	119 773.75	69 203.530	7297.237	13 915.827	16 625.058
Spline (distance) with knot at 10 km + household	119 740.49	69 190.891	7299.235	13 917.700	16 621.391
Spline (distance) with knot at 15 km	119 787.96	69 212.733	7296.504	13 915.688	16 627.322
Spline (distance) with knot at 15 km + household	119 743.05	69 193.487	7298.503	13 917.576	16 621.453
Spline (distance) with knot at 20 km	119 796.09	69 217.593	7296.408	13 915.635	16 628.180
Spline (distance) with knot at 20 km + household	119 743.91	69 194.279	7298.408	13 917.543	16 621.327
Spline (distance) with knot at 25 km	119 802.40	69 221.326	7296.454	13 915.762	16 628.445
Spline (distance) with knot at 25 km + household	119 744.67	69 194.883	7298.449	13 917.709	16 621.318
Natural log of distance	119 789.07	69 207.654	***7294.896***	***13 913.204***	16 626.328
Natural log of distance + household	***119 734.75***	***69 184.486***	7296.866	13 915.077	16 620.510

a Best fit model is given in bold and italics.

**Fig. 2.**
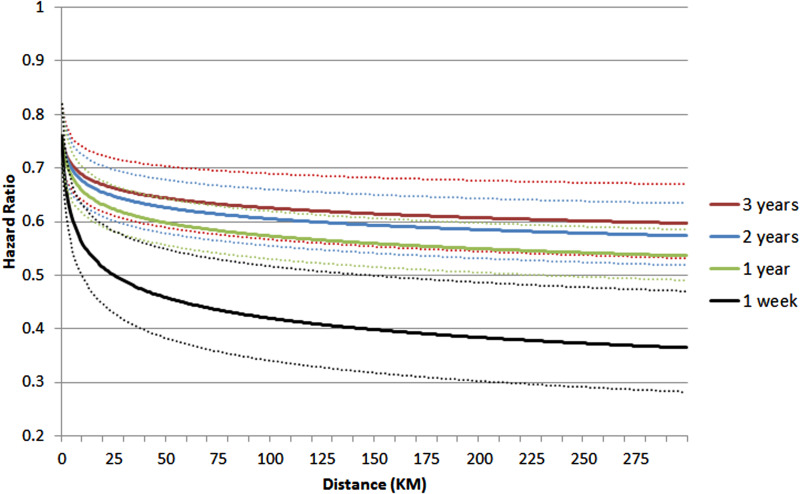
The HR (±95% CI) for a first registration of DA in S2 in 3 years after a first registration for DA in S1 as a function of distance (in kilometres) they were residing apart at the time of S1's DA and the time period from D1's first DA registration. For details and covariates, see Appendix Table A3.

Risk for DA in S2 was greater the younger the age at S1's registration, the smaller the S1–S2 age difference and the greater the DA density in the SAMS area where S2 was residing. Of note, as seen in Fig. 1, S2's risk for DA does not begin at 1.0 because of the household effect. Significant interactions were seen between both the household and proximity effects, and time since S1's registration, indicating a failure of the proportionality assumption. For example, the HR of log of kilometres in the prediction of risk for DA registration in S2 at 1 week, 1 month, 1, 2 and 3 years after DA registration in S1 was estimated to equal, respectively, 0.88 (0.84; 0.92), 0.94 (0.93; 0.96), 0.95 (0.93; 0.97) and 0.96 (0.94; 0.98) (Appendix Table A1 and Fig. 2).

Details about the informative male–male, female–female, female–male and male–female sibling pairs for proximity analysis of DA are also seen in Table 1. The best-fit model for male–male pairs is the same as that seen for the entire sample (household  + log of distance). For female–female and female–male pairs, the best fit model was log of distance only although the fit improvement over the covariate only model was slight. For male–female pairs, the best fit model included only a household effect. Proximity effects for male–male pairs (HR  =  0.93, 0.91; 0.95) were slightly stronger than seen with all pairs, while effects for the female–female and female–male pairs were weaker and not significant.

We identified 2470 informative sibships for DA where the best-fit model was the same as that obtained in the sibling pairs. The HR for distance was slightly stronger in the sibships than in the pair analyses (Table 3).

**Table 3.** Results from Cox proportional hazards models (±95% CI) on full sibling pairs and sibships from the Swedish population where at least one in the pair are registered for DA^a^

		HRs (±95% CI)				
	Design	All	Male to male	Female to female	Female to male	Male to female
Not same household	Sib pairs	0.76 (0.71; 0.82)	0.80 (0.73; 0.88)	–	–	0.73 (0.72; 0.87)
Natural log of distance		**0.94 (0.92; 0.95)**	**0.93 (0.91; 0.95)**	**0.96 (0.91; 1.01)**	**0.97 (0.94; 1.01)**	–
Age at registration		0.95 (0.94; 0.95)	0.94 (0.94; 0.95)	0.95 (0.94; 0.96)	0.94 (0.93; 0.95)	0.96 (0.95; 0.97)
Age difference		0.92 (0.91; 0.93)	0.92 (0.90; 0.93)	0.91 (0.87; 0.96)	0.92 (0.88; 0.95)	0.93 (0.89; 0.96)
SAMS density		1.08 (1.07; 1.09)	1.08 (1.06; 1.09)	1.09 (1.07; 1.12)	1.11 (1.09; 1.13)	1.08 (1.06; 1.09)
Not same household	Sibships	0.61 (0.53; 0.69)				
Natural log of distance		**0.90 (0.87; 0.93)**				
Age difference		0.95 (0.93; 0.96)				
SAMS density		1.15 (1.12; 1.18)				

a Key results for log of distance are given in **bold**. For the ‘all’ analyses, sex of S1 and S2 were controlled for.

### Examining registry effects for DA

Could registry effects bias upwards our estimates of proximity effects for DA if police or physicians were more likely to, respectively, arrest or treat siblings for DA who lived close together? We explored four registry based patterns of DA transmission from S1 → S2: medical → medical, criminal → criminal, criminal → medical and medical → criminal (Appendix Table A2). These did not significantly differ for either household or proximity effects (Appendix Table A3).

## Discussion

We utilised information in Sweden on the geographical location of individuals to study the psychological transmission of DA, postulating, in accord with empirical evidence (Suggs, 1989; Lee *et al*., 1990; White and Riedmann, 1992; Stocker and Lanthier, 1997; White, 2001; Spitze and Trent, 2006; Van Volkom, 2006; Mulder and van der Meer, 2009), that sibling proximity in adulthood reflects both frequency of contact and emotional closeness. We controlled for a key potential confound – that the effect of sibling proximity might arise simply from correlated psychosocial risk for siblings living closer together. We did so by accounting for, in our Cox models, the density of DA in similarly aged individuals in the small community in which S2 resided at the time of S1's registration. The density of DA effectively controls for most community factors that impact on S2's risk for DA as well as any possible ascertainment biases in DA rates.

Furthermore, we sought to increase confidence in our conclusions by two further analyses. First, did time since S1 registration interact with proximity effect on risk in S2? Detecting such an effect supports the validity of our findings because such results could not plausibly arise from confounding community influences, as these would not change substantially over the short time periods examined. Second, sibling pairs share a wide range of familial risk factors – such as rearing environment, peer groups and social class – that could confound our analyses. We utilised a sibship design that compared risks for registration in multiple siblings of S1 as a function of their relative proximity to S1. In these analyses, all comparisons were within the same family, thereby controlling for many background, genetic, family and community environmental factors.

Transmission within social networks or ‘social contagion’ (Christakis and Fowler, 2013) has been demonstrated for both alcohol intake (Rosenquist *et al*., 2010) and smoking (Christakis and Fowler, 2008). Most contagion models for DA have been developed within adolescent peer group or community settings and examine only illicit substance use (Dinger and Oetting, 1993; Bauman and Ennett, 1994; Galea *et al*., 2004; Anthony, 2006; Dishion and Dodge, 2006; Ennett *et al*., 2006). Peer group nominations, which are critical to many such analyses, have potential biases (Liebow *et al*., 1995). We are unaware of prior efforts to examine transmission of DA in siblings, when the key predictor variable – geographical proximity – is objectively defined and the analysis focuses on DA rather than quantity of use.

Five additional analyses supported the validity of our DA findings. First, transmission of DA was stronger in sibling pairs closer together in age. Second, DA transmission was substantially stronger in same-sex *v*. opposite-sex sibling pairs. Third, controlling for familial effects in our sibship analyses, proximity effects were not attenuated. Fourth, the strength of the proximity effect was more robust immediately after S1's registration and this effect attenuated over time. Fifth, registry specific effects, which might result from police or physicians being more likely to arrest or treat siblings for DA who live close together, do not appear to contribute substantially to our findings. We also performed an additional validity check on our results. Could we confirm prior evidence that older siblings have a particularly strong influence on drug use in their younger siblings? (Brook *et al*., 1983; Needle *et al*., 1986; Sakai *et al*., 2004; Kendler *et al*., 2013*b*). We indeed found this to be the case. A model controlling for household effect, log of distance, age difference, age of S1 at registration and SAMS density, the HR for DA in S2, given that S2 was younger *v*. older than S1, was 1.70 (1.60; 1.80).

The transmission of DA among siblings might involve S1 changing attitudes about drug use in S2 as well as providing a positively perceived model whose behaviour could be imitated (Brook *et al*., 1983). However, in addition, transmission of DA could include information about sources of supply and the direct transfer of the abused substance.

### Prior relevant study on DA

Our findings on within-family transmission of DA based on precise proximity measures are consistent with findings from our prior analyses dividing parent–offspring, sibling and cousin pairs into three proximity categories: household, community and metropolitan area (Kendler *et al*., 2019). In those analyses, the age of at-risk secondary cases was markedly restricted (to ages 19–23) to maximise the proportion of cohabiting parent–offspring and sibling pairs while no such restrictions were operative in our present analyses. Our results confirmed and extended our earlier findings for DA showing evidence for psychological transmission of DA among relatives (with transmission strongest for cohabiting relatives and weakest in those only residing in the same metropolitan area). In accord with our results, our prior analyses also found, in both parent–offspring and cousin pairs, stronger DA transmission in male–male pairs and in those closer in age (Kendler *et al*., 2019).

The sibling pairs used in our prior analyses constituted 16.9% of our sample examined here. To determine whether the similarity of results arose from sample overlap, we repeated our main analyses without those pairs. As seen in Appendix Table A4, no appreciable changes were seen in our key results. For example, the HR for the log of distance in the sibling pairs was 0.94 (0.92; 0.95) in our entire sample and 0.94 (0.92; 0.96) in our analyses dropping the pairs utilised in the prior analyses thereby confirming that this report indeed represented an independent and methodologically quite different confirmation of our previous findings.

### Limitations

These results should be interpreted in the context of six potential methodological limitations. First, our results apply only to the Swedish population assessed between 1975 and 2012. Electronic means of human communication have evolved dramatically across this period and may change the role of geographical proximity in transmission of DA. Second, our assessment of DA was limited to data available from Swedish registries. While such administrative data have important advantages (e.g. no refusals or reporting biases), it surely results in false negative reports. Our measures likely reflect the more severe end of DA that results in medical or legal consequences. Third, DA is an emerging phenomenon and the timing of registration is unlikely to capture precisely the onset of abuse. This is one reason why we used a 3-year window to assess transmission of DA registration within siblings, the second reason being we observed most within-family transmission of DA within this limit in a prior Swedish study (Kendler *et al*., 2019). Fourth, our measure of distance between siblings was substantially skewed, hence our use of a logarithmic transformation. As an additional attempt to examine possible biases, we repeated our key analysis excluding the largest 1, 5 and 10% of inter-sibling distances with no appreciable change in parameter estimation. Fifth, we cannot rule out the possibility that transmission of risk to S2 might be influenced by onsets of DA in peers of S1. This is unlikely because such onsets would need to be tightly correlated in time with onsets in S1 to explain the stronger effects of distance on S2 risk shortly after S1's onset.

Furthermore, S2 is likely be in closer touch with and have a stronger emotional bond with his sibling than with his sibling's peers and peer effects would be unlikely to produce our strong evidence for stronger DA transmission among same-sex siblings. Finally, attribution of causal effects in non-experimental data is always fraught with hazard. While our results are consistent with a direct causal effect of DA in S1 and risk for DA in S2, and we have attempted to control for key relevant confounders, skepticism about such causal claims is warranted.

## Conclusion

In Sweden, the probability of transmission of DA registration from a young adult to his or her sibling is stronger the shorter the distance between their places of residence. This relationship is best described by a logarithmic function. The probability of transmission falls rapidly over relatively short distances asymptoting at between 100 and 150 km. A range of analyses support the validity of these primary results. However, as with all observational epidemiological investigations, causal inference should be regarded as tentative. If correct, our findings, in line with our previous research (Kendler *et al*., 2012, 2013*a*, 2013*b*, 2015*a*), suggest that the transmission of DA within families reflects a complex mixture of genetic and environmental effects. Some of the environmental effects on DA appear to act during childhood and adolescence, probably reflecting the influences of the home and of peer groups. We here show that environmental/psychological factors as sources of social contagion continue to play an important role in early adulthood. Our findings also have clinical relevance. After the onset of DA, the affected individual's siblings living nearby are at high risk and may constitute good targets for rapidly employed prevention efforts.

## Data

Data used in these analyses are not publicly available due to confidentiality concerns of the Swedish

authorities.

## Appendix

**Table A1.** Test of the proportionality assumption for the Cox proportional hazards models on full sibling pairs from the Swedish population where at least one in the pair are registered for DA

	DA	
	Not same household	Log of distance
Log of time × household (*p*-value for the interaction term)	0.021	
Log of time × log of distance (*p*-value for the interaction term)		0.0021
Illustration of the HR at different time points		
1 week	0.62 (0.52; 0.75)	0.88 (0.84; 0.92)
1 year	0.77 (0.71; 0.83)	0.94 (0.93; 0.96)
2 years	0.80 (0.73; 0.87)	0.95 (0.93; 0.97)
3 years	0.81 (0.74; 0.89)	0.96 (0.94; 0.98)

Numbers are HRs and 95% confidence intervals (CIs). All pairs are included in the analyses.

**Table A2.** Descriptive statistics on full sibling pairs from the Swedish population where at least one in the pair are registered for DA

	DA			
	Medical to medical	Criminal to criminal	Criminal to medical	Medical to criminal
*N*	68 386	84 908	84 908	68 386
% in S2	1.0	3.8	0.7	1.0
S2 affected				
Distance (km) 25th-50th-75th	1-5-45	0-1-13	0-3-29	0-4-30
Distance (km) mean	66.6	37.4	53.2	52.3
Same household	19.4%	40.1%	29.0%	27.8%
Age at registration S1	28.4	23.0	25.5	25.4
Age difference	3.6	3.4	3.5	3.5
YoB S1, mean, s.d.	1968	1981	1979	1978
YoB S2, mean, s.d.	1968	1981	1978	1978
SAMS density S1 (±5)	1.5	3.2	2.5	1.7
SAMS density S2(±5)	1.3	3.0	1.6	2.1
S2 not affected				
Distance (km) 25th-50th-75th	3-13-79	1-7-47	1-6-45	3-13-79
Distance (m) mean	84.6	69.2	68.1	84.7
Same household	9.9%	20.7	21.3%	9.8%
Age at registration S1	32.1	27.2	27.0	32.1
Age difference	4.2	4.0	4.0	4.2
YoB S1, mean, s.d.	1966	1978	1978	1965
YoB S2, mean, s.d.	1965	1977	1977	1965
SAMS density S1 (±5)	1.2	2.4	2.4	1.2
SAMS density S2(±5)	0.7	1.8	0.9	0.6

**Table A3.** Results from Cox proportional hazards models on full sibling pairs from the Swedish population where at least one in the pair are registered for DA

	HRs and 95% CIs				
	Medical to medical	Criminal to criminal	Criminal to medical	Medical to criminal	Test of heterogeneity
Not same household	0.83 (0.65; 1.06)	0.79 (0.72; 0.86)	0.90 (0.71; 1.13)	0.66 (0.53; 0.81)	*p*-value: 0.2244
Log of distance	0.95 (0.91; 0.99)	0.93 (0.91; 0.95)	0.96 (0.92; 1.01)	0.96 (0.92; 1.00)	*p*-value: 0.3651
Age at registration	0.97 (0.96; 0.98)	0.95 (0.94; 0.95)	0.99 (0.98; 1.00)	0.93 (0.92; 0.95)	
Age difference	0.91 (0.88; 0.95)	0.92 (0.91; 0.94)	0.92 (0.88; 0.96)	0.93 (0.89; 0.96)	
SAMS density	1.09 (1.07; 1.12)	1.08 (1.07; 1.09)	1.14 (1.11; 1.17)	1.22 (1.20; 1.24)

**Table A4.** Sib pair analyses (Table 3) repeated excluding individuals from prior study of ‘A contagion model for within-family transmission of drug abuse’

	HRs and 95% CIs				
	All	Male to male	Female to female	Female to male	Male to female
DA					
Not same household	0.70 (0.64; 0.77	0.73 (0.65; 0.82)	–	–	0.66 (0.53; 0.82)
Log of distance	0.94 (0.92; 0.96)	0.93 (0.91; 0.95)	0.95 (0.90; 1.01)	0.97 (0.94; 1.01)	–
Age at registration	0.95 (0.94; 0.96)	0.95 (0.94; 0.95)	0.95 (0.94; 0.96)	0.94 (0.93; 0.95)	0.98 (0.96; 0.99)
Age difference	0.92 (0.90; 0.93)	0.91 (0.90; 0.93)	0.91 (0.86; 0.96)	0.92 (0.88; 0.95)	0.93 (0.89; 0.97)
SAMS density	1.08 (1.07; 1.09)	1.08 (1.06; 1.09)	1.09 (1.06; 1.12)	1.11 (1.09; 1.13)	1.08 (1.06; 1.10)
